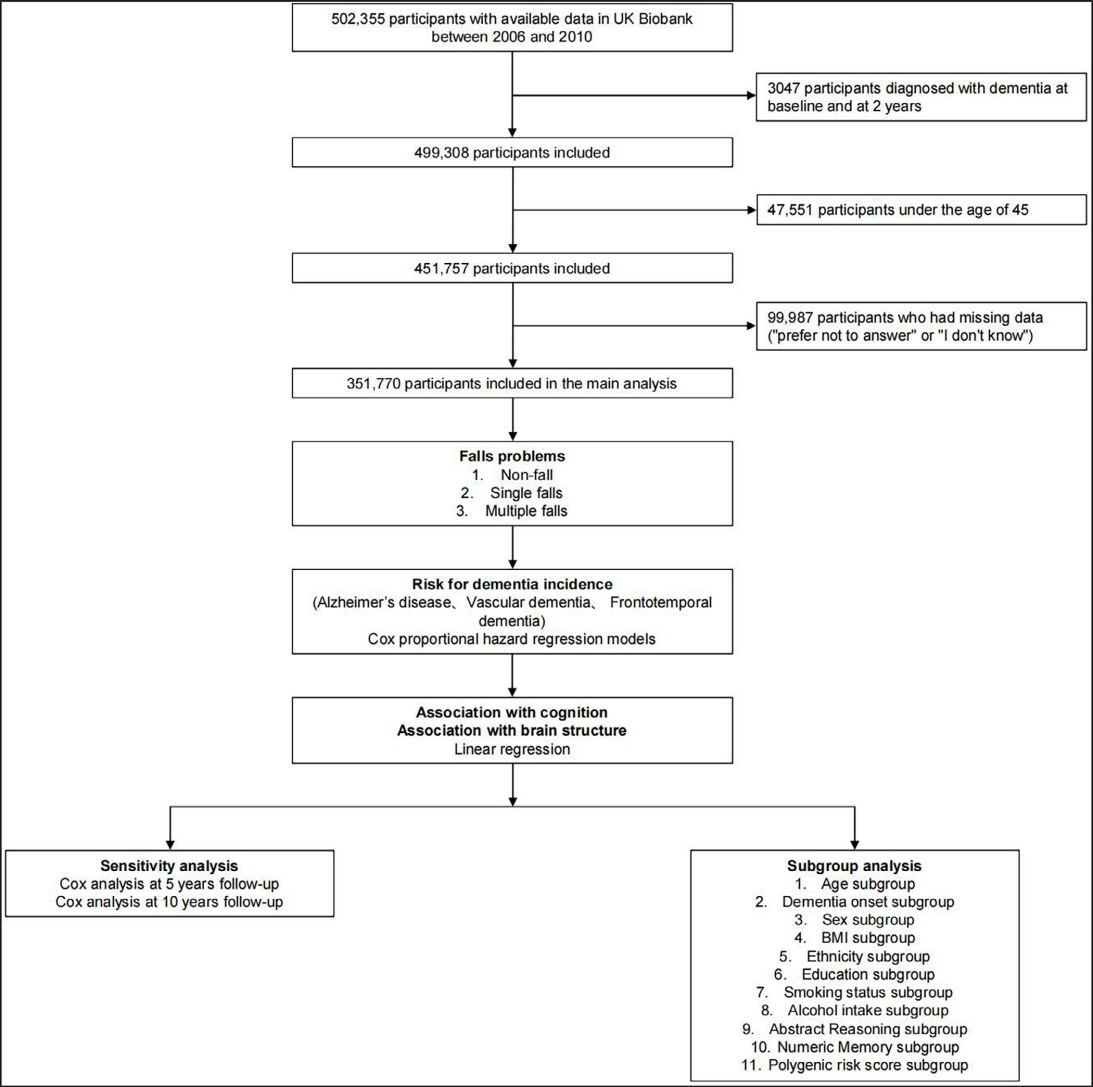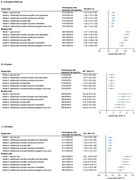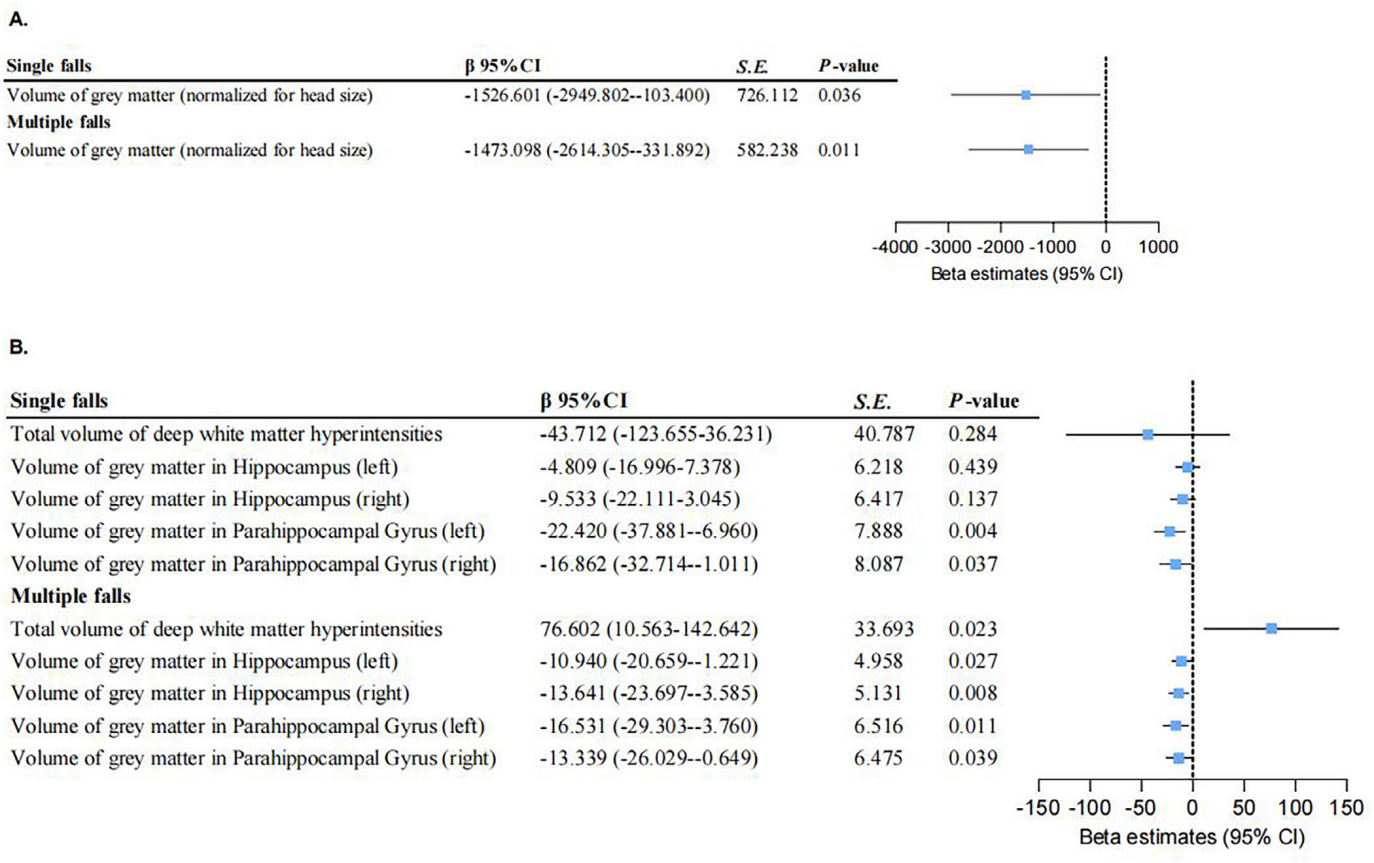# Associations of falls and genetic risk with dementia risk: a prospective cohort study from UK biobank

**DOI:** 10.1002/alz70860_102423

**Published:** 2025-12-23

**Authors:** Aonan Li, Shaojiong Zhou, Jie Chang, Tao Wei, Bo Zhao, Yiwei Zhao, Yi Xing, Yi Tang

**Affiliations:** ^1^ Department of Neurology & Innovation Center for Neurological Disorders, Xuanwu Hospital, Capital Medical University, National Center for Neurological Disorders, Beijing, Beijing, China; ^2^ National Center for Neurological Disorders, Xuanwu Hospital, Capital Medical University, Beijing, Beijing, China; ^3^ Department of Neurology & Innovation Center for Neurological Disorders, Xuanwu Hospital, Capital Medical University, National Center for Neurological Disorders, Beijing, Beijing, China; ^4^ Neurodegenerative Laboratory of Ministry of Education of the People's Republic of China, Beijing, Beijing, China

## Abstract

**Background:**

The relationship between falls and dementia risk remains unclear. This study aims to investigate this association and assess the impact of genetic risk on this relationship.

**Method:**

We included 351,770 participants from the UK Biobank who were free from dementia at baseline. A Cox model was used to estimate the relationship between falls and dementia risk.

**Result:**

After a median follow‐up of 13.6 years, 5,821 developed dementia. Compared to individuals without a history of falls, individuals with single and multiple falls had hazard ratios (HRs) for dementia of 1.24 (95% CI, 1.16–1.33) and 1.79 (95% CI, 1.64–1.94), respectively. This risk was more pronounced in individuals with better cognitive function at baseline. Those experienced single falls demonstrated reduced whole‐brain gray matter volume (β = −1526.601, *p* = 0.036) and parahippocampal gyrus volume (β_left_ = −22.420, *p* = 0.004; β_right_ = −16.862, *p* = 0.037), whereas those experienced multiple falls, in addition to these findings, demonstrated increased deep white matter hyperintensities (β = 76.602, *p* = 0.023) and decreased gray matter volume in the hippocampus (β_left_ = −10.940, *p* = 0.027; β_right_ = −13.641, *p* = 0.008). Furthermore, falls and polygenic risk scores showed a significant interaction (*p* = 0.001), with higher dementia risk observed in individuals with multiple falls and high genetic risk.

**Conclusion:**

Falls are an early predictor of dementia risk, and genetic susceptibility facilitates this association. Falls indicate potential atrophy in cognition‐related brain regions. Falls are a simple and easily observable sign for dementia screening.